# DNA double-strand break formation and repair as targets for novel antibiotic combination chemotherapy

**DOI:** 10.2144/fsoa-2019-0034

**Published:** 2019-09-02

**Authors:** Vincent Amarh, Patrick K Arthur

**Affiliations:** 1 Department of Biochemistry, Cell & Molecular Biology, West African Center for Cell Biology of Infectious Pathogens, University of Ghana, PO Box LG54, Legon, Accra, Ghana

**Keywords:** antibiotic combination chemotherapy, cell-based assays, DNA double-strand break, genome stability, homologous recombination, nonhomologous end joining, quinolone resistance, ribosome inhibitors, SOS response, type II topoisomerase

## Abstract

An unrepaired DNA double-strand break (DSB) is lethal to cells. In bacteria, DSBs are usually repaired either via an error-prone pathway, which ligates the ends of the break or an accurate recombination pathway. Due to this lethality, drugs that induce persistent DSBs have been successful in bacterial infection treatment. However, recurrent usage of these drugs has led to emergence of resistant strains. Several articles have thoroughly reviewed the causes, mechanisms and effects of bacterial drug resistance while others have also discussed approaches for facilitating drug discovery and development. Here, we focus on a hypothetical chemotherapeutic strategy that can be explored for minimizing development of resistance to novel DSB-inducing compounds. We also highlight the possibility of utilizing bacterial DSB repair pathways as targets for the discovery and development of novel antibiotics.

## Lay abstract

Our health systems face a huge challenge in the form of antimicrobial resistance, which may result in many common infections becoming untreatable. The same antibiotics that gave modern medicine its power are fast losing their hold on the germs that cause disease. Many options are being developed to restore the control that antibiotics have on the microbes that cause many diseases. In this perspective, we outline a concept that is built around the way and manner in which bacteria mend their DNA whenever there is a break in the DNA chain. We discuss the merits of finding a new class of drugs that obstruct bacterial ability to mend their broken DNA. In this scenario, a combination of these new drugs with existing drugs or other new drugs that cause breaks in bacterial DNA would become a powerful therapeutic regimen. This concept, when fully developed, will offer hope in our effort to combat antimicrobial-resistant infections.

## Antibiotic resistance

Antibiotics have been, and still are, instrumental in the global treatment and control of bacterial infections [1–3]. They have also boosted medical interventions, such as surgeries, for which improvement would otherwise have stagnated due to complications arising from bacterial infections. Thus, antibiotics are indispensable for sustaining quality global health via eradication of infections caused by bacterial pathogens. It was therefore not surprising for the WHO to indicate that the increasing prevalence of multidrug-resistant bacterial strains is a primary threat to global public health [4]. These multidrug-resistant strains have rendered several natural products with antimicrobial activities obsolete in clinics [5]. However, some of these obsolete antibiotics that had lost their efficacy against pathogenic bacteria have been resuscitated for use in clinics via chemical synthesis of derivatives of the active compound [6,7]. Incidentally, these second-, third- and fourth-generation drug derivatives are gradually losing their usefulness in clinics too due to the development of resistance against their antimicrobial activity.

**Figure 1.**
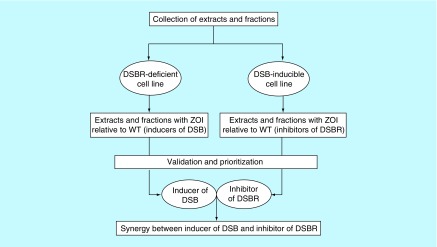
Cell-based screening strategy for development of drugs targeting DNA double-strand break formation and repair in bacteria. A library of extracts (or fractions) are tested simultaneously against a DSBR-deficient cell line (e.g., a *recB* mutant of *Escherichia coli*) and a DSB-inducible cell line (e.g., the *E. coli* SbcCD/palindrome system [9]). A WT *E. coli* cell line, which is unable to generate the inducible DSB, serves as a control for both screening assays. Extracts (or fractions) that exhibit ZOI against the DSBR-deficient cell line, but not the WT, are designated as inducers of DSBs. Similarly, extracts (or fractions) that exhibit ZOI against the DSB-inducible cell line, but not the WT, are designated as inhibitors of DSBR. The synergistic effect of these candidate extracts (or fractions) is validated by testing in unison against the WT cell line alone. The combination of inducers of DSBs and inhibitors of DSBR that generates ZOI against the WT cell line is expected to exhibit novel antimicrobial activity against pathogenic strains of *E. coli*. This cell-based screening model is also applicable to drug development against other pathogenic bacterial strains. DSB: Double-strand break; DSBR: DSB repair; WT: Wild-type; ZOI: Zone of inhibition.

**Figure 2.**
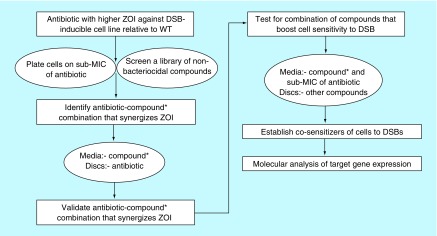
A model for enhancing the sensitivity of cells to inhibitors of DNA double-strand break repair. Antibiotics that exhibit higher zones of inhibition against the DSB-inducible cell line relative to wild-type are predicted to mediate inhibition of DSBR, thereby sensitizing cells to DSB formation. In order to boost this unique activity of the antibiotic, a library of nonbactericidal compounds is screened against the two cell lines following exposure to sub-MIC of the antibiotic. The nonbactericidal compounds that cause a further increase in the zones of inhibition are validated via a disc diffusion assay in which the agar plate is amalgamated with the compound and the antibiotics are infused onto discs. The procedure is repeated to demonstrate combinations of nonbactericidal compounds that exacerbate the sensitivity of cells to DSB formation in the presence of the antibiotic. Analysis of the expression profile of *DSBR* genes and associated stress response genes could provide valuable insight on the molecular mechanism underlying co-sensitization of cells to DSB formation. Importantly, the model could be incorporated into the cell-based screening assay, described in Figure 1, to identify extracts containing very low concentrations of inhibitors of DSBR. *Nonbactericidal compound. DSB: DNA double-strand break; DSBR: DSB repair; MIC: Minimum inhibitory concentration; ZOI: Zone of inhibition.

The historic antibiotics, such as penicillin, streptomycin, chloramphenicol and tetracycline, were originally isolated from fungal sources or soil bacteria [8]. Since fungal and soil bacterial species are both diverse in nature, an enormous amount of research is currently being invested into discovering novel antibiotics from these natural sources. It is anticipated that these novel compounds might exhibit antimicrobial activity against drug-susceptible pathogenic bacterial strains and strains that are resistant to antibiotics used for first-line treatments. One underlying question still remains: is the search for novel broad-spectrum antimicrobial compounds sufficient to helps us with the menace originating from bacterial resistance to the available antibiotics? A new phenotypic screening approach that goes beyond the paradigm growth inhibitory assays should be explored for development of novel drugs endowed with a unique mechanism of action. The rationale would be to systematically develop a set of cell lines that allows for the detection of new classes of bioactive compounds that target specific and essential cellular processes. The use of the paradigm cell-based assays is likely to continue to yield the same classes of antibiotics against the same range of targets for which resistance mechanisms already exist or have evolved. An alternative strategy would be to design cell-based and target-specific assays that detect new classes of compounds that act synergistically to inhibit the development of resistance. A pragmatic model of such an assay, which relies on the lethality of an unrepaired DNA double-strand break (DSB), is shown in Figure 1. The problems associated with screening of natural products are the high dynamic range of compounds, in terms of chemical diversity and concentration. To address these issues, the new cell-based and target-specific assay ought to include strategies that improve on selectivity and sensitivity to many orders of magnitude. An example of a predictive approach for enhancing the sensitivity of cells to inhibitors of bacterial DNA double-strand break repair (DSBR) is illustrated in Figure 2.

## Bacterial genome stability as a target for antibiotics

Cell wall synthesis, protein synthesis, nucleic acid synthesis and genomic DNA integrity are usually the main cellular targets of antibiotics in bacterial pathogens [10–14]. Quinolones are examples of antibiotics that compromise the stability of bacterial genomic DNA.

The lethality of unrepaired DSBs underlies the antimicrobial activity exhibited by the quinolones [10,15–17]. Quinolones bind to the active site of the bacterial type II topoisomerases, following DNA cleavage, to form a quinolone–enzyme complex, which perturbs re-ligation of the cleaved DNA [18]. This cascade of events leads to accumulation of DSBs in bacteria that are exposed to quinolones. DNA cleavage by the type II topoisomerases is a necessity for releasing the torsional stress that accumulates within the chromosome during DNA replication [19]. Consequently, exploiting the function of these bacterial type II topoisomerases to generate lethal DNA damage made quinolones very effective against a wide variety of bacterial infections [6].

## Resistance of pathogenic bacteria to quinolones

Even though quinolones have been used as effective antibiotics, resistant strains have gradually emerged within the last half century [20]. Resistance to quinolones typically arises via mutations in the genes encoding the DNA gyrase and DNA topoisomerase IV enzymes [21].

Efflux of quinolones from the bacterial cell and the acquisition of plasmids, which encode quinolone resistance genes, have also been reported as secondary mechanisms that are utilized by many pathogenic bacteria to confer resistance against quinolones [20]. Surveillance data have also shown that high prevalence of quinolone resistance occurred during increased usage of ciprofloxacin, which is one of the second generations of the quinolone drugs [20,22]. These observations indicate the need to screen for new molecules with different mechanisms of inducing DSBs. Ideally, the mode of action of these novel antimicrobial molecules should not be dependent on topoisomerase- or gyrase-mediated DNA cleavage. Strategies that could minimize development of resistance to these new molecules must also be considered during the initial phase of design of these molecules into drugs.

## Novel compounds targeting stability of bacterial genomic DNA

**Figure 3.**
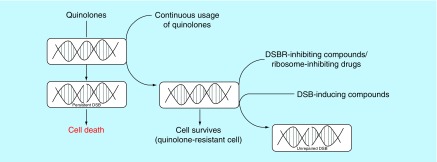
Cell-based approach for development of combination chemotherapy targeting DNA double-strand break formation and inhibition of repair. Quinolones generate persistent DSBs in bacteria and eventually cause cell death. The continuous usage of quinolones for treatment of bacterial infections has resulted in the emergence of strains which are resistant to the drug. A plausible approach for treatment of infections caused by quinolone-resistant strains is to administer compounds capable of inhibiting bacterial DSB repair in combination with novel DSB-inducing compounds. Alternatively, drugs that inhibit the bacterial ribosomes could be used to deplete global protein synthesis, including DSB repair proteins, thereby leading to accumulation of unrepaired DSBs in the quinolone-resistant strains. DSB: DNA double-strand break; DSBR: DNA double-strand break repair.

In the quest to identify new drug candidates that compromise the stability of bacterial genome, it is preferable to screen for novel compounds that generate DSBs and administer in combination with compounds that inhibit the concomitant repair event (Figure 3). An advantage of this chemotherapeutic approach is the increased sensitivity of bacterial pathogens to low doses of DSB-inducing drugs due to the effect of the DSBR inhibitors. Consequently, adverse effects caused by high drug dosage would be circumvented. For example, perturbation of the human gut microbiome during prolong antibiotic chemotherapy would be minimized by this combination chemotherapeutic approach [23]. The development of drug resistance due to exposure of bacterial pathogens to high dose of DSB-inducing drugs might also be minimized by the proposed chemotherapeutic approach.

The DSBR pathway is essential for cell viability because it repairs spontaneous DSBs that are generated during normal cellular metabolism and growth, as well as DSBs induced by exogenous agents. Compounds that are capable of inhibiting DSBR are less likely to be efficient drugs when administered alone because the frequency of spontaneous (uninduced) DSB formation per cell cycle is very low in individual bacterial cells [24]. DSBR-inhibiting compounds could also boost chemotherapy by increasing the susceptibility of pathogenic bacteria to drugs that induce DSBs. Incidentally, compounds that inhibit DSBR in bacteria are yet to be discovered and the key strategy for success will be a cell-based assay using cell lines that have inducible DSB-generating genetic elements.

## DNA double-strand break repair pathways in bacteria

In bacteria, DSBs are mostly repaired by either the homologous recombination pathway or the nonhomologous end joining (NHEJ) pathway. In *Escherichia coli*, DSBs are repaired by homologous recombination using an undamaged DNA template [25], which is usually the sister chromosome that is obtained from chromosomal replication. Even though the key proteins used for NHEJ (Ku and Ligase D) are yet to be discovered in *E. coli*, an end-joining activity for repairing DSBs has been reported [26]. The alternative end-joining repair mechanism was shown to be dependent on the activity of the replicative Ligase A. Other bacteria such as *Staphylococcus aureus, Clostridium perfringens, Neisseria meningitidis* and *Mycobacterium leprae* might rely predominantly on homologous recombination for repair of DSBs since they also lack the Ku and Ligase D proteins [27]. In contrast, *Bacillus* sp., *Mycobacterium tuberculosis*, *Streptomyces coelicolor*, *Pseudomonas* sp*.* and *Xanthomonas* sp*.* encode functional Ku and Ligase D proteins, which might suggest that these organisms are capable of repairing DSBs via the NHEJ pathway [27,28]. In fact, previous studies have demonstrated that *Bacillus subtilis* and *Mycobacterium smegmatis* use the NHEJ pathway for repairing DSBs that are generated during the dormant phase of these organisms [29,30]. Besides the homologous recombination and NHEJ pathways, repair of DSBs via the single-strand annealing (SSA) pathway has been reported for *M. smegmatis* [31]. SSA was initially shown to be used for repairing a DSB, which is flanked by homologous DNA repeat sequences in *Saccharomyces cerevisiae* [32]. It would be critical to determine which of the two major DSBR pathways is vastly affected during the search for inhibitors (Figure 1). A possible approach is to use a *recA* mutant of *M. smegmatis* which, unlike *E. coli*, encodes a well-defined Ku and Ligase D that mediate NHEJ. In-depth analysis of DSB repair in a *recA* deletion mutant of *M. smegmatis* can provide insight on the relevance of NHEJ in development of antibacterial-resistant strains; NHEJ is an error-prone pathway for repairs of DSBs [33].

## Boosting susceptibility of bacteria to DSBs by inhibiting recombinational repair

In bacteria, DSBR by homologous recombination has been studied extensively using *E. coli* as a model organism. Recombinational repair of DSBs in *E. coli* is initiated by binding of the RecBCD enzyme complex to each end of the DSB. The RecBCD complex processes the ends of the DSB to generate ssDNA, unto which the same enzyme complex loads monomers of the RecA protein to form a nucleoprotein filament [34,35]. The RecA nucleoprotein filament catalyses the search for the undamaged homologous DNA and the subsequent strand invasion reaction, which are fundamental to genetic recombination [36,37]. The strand invasion reaction enables re-synthesis of the DNA sequences that were lost at the site of the DSB [38]; loss of DNA at the site of the DSB occurs by the action of RecBCD complex during the initial phase of DSBR [34], and by the activity of the ubiquitous exonucleases in the cell. The completion of DNA repair synthesis and the resolution of Holliday junctions by the RuvABC enzyme complex result in the formation of two intact duplex DNA that are separated from each other [39,40]. The key proteins used for repair of DSBs by homologous recombination are dissimilar in bacteria and humans, hence they represent potential targets that could be exploited to selectively enhance the sensitivity of pathogenic bacteria to DSB-inducing drugs.

For organisms that repair DSBs predominantly by homologous recombination, such as *E. coli*, inhibition of recombinational repair renders these bacteria to be extremely sensitive to DSBs [9,40–42]. Unlike *E. coli*, the diverse pathways for repairing DSBs in organisms such as *B. subtilis* and *M. smegmatis* present limitations for exploiting bacterial DSBR as target for enhancing the susceptibility of these organisms to drugs that generate DSBs. Specifically, potential drug candidates capable of inhibiting only one of the repair pathways would not render *B. subtilis* and *M. smegmatis* to be very sensitive to DSBs. Nonetheless, if the bacterial NHEJ mechanism is primarily used for DSBR during the dormant phase of the cell cycle [43], due to absence of a homologous DNA template, inhibition of recombinational repair could render actively growing cells of *B. subtilis* to be sensitive to DSB formation. The existence of a third distinct DSBR mechanism (SSA pathway) in *M. smegmatis* [31] implies that a combination of compounds might be required to robustly inhibit repair of DSBs in this organism. Hence, the chemotherapeutic strategy of inhibiting DSBR in order to increase the susceptibility of bacterial pathogens to DSB-inducing drugs is intricate for *M. smegmatis*. This problem can be addressed by using mutant cell lines of *M. smegmatis* that are deficient in either the Ku and Ligase D or the SSA pathway to assess the extent to which they contribute to the repair of DSBs induced by compounds detected in natural product screens. The empirical evidence of this nature will direct the forward design of DSBR inhibitory targets necessary for improving the efficacy of new DSB-inducing compounds or the current fluoroquinolones.

## Combination of ribosome inhibitors with DSB-inducing drugs to boost chemotherapy

In addition to the fluoroquinolones, ribosome inhibitors are also one of the successful antibiotics that have been used for the treatment of bacterial infections. Ribosome inhibitors are diverse in chemical structure and they target components of either the small (30S) or large (50S) subunit of the bacterial ribosome, ultimately inhibiting protein synthesis [44]. Erythromycin, chloramphenicol, clindamycin and linezolid inhibit the 50S subunit of bacteria whereas tetracycline, streptomycin, kanamycin and gentamicin are inhibitors of the 30S ribosomal subunit [45]. Chloramphenicol inhibits the bactericidal activity of nalidixic acid, indicating that protein synthesis is a requirement for the antibacterial activity of the quinolone [46]. Subsequent studies have demonstrated that fluoroquinolones such as ofloxacin can exhibit bactericidal activity in the absence of protein synthesis [47,48]. These fluoroquinolones require the DNA gyrase subunit A to exhibit bactericidal activity in the absence of protein synthesis; *nalA* mutants of *E. coli* are not sensitive to ciprofloxacin and ofloxacin in the absence of protein synthesis [49].

The Gram-negative *E. coli* is highly sensitive to very low concentrations of fluoroquinolones in comparison to the nonfluorinated quinolones, such as nalidixic acid [50]. Below the optimum bactericidal concentration of fluoroquinolones, the bacteria undergo extensive filamentation due to induction of the DNA stress response (SOS response) [51]; the SOS response controls expression of several proteins that are essential for repair of DNA damage [52]. The lethality, which occurs at the optimum bactericidal concentration of fluoroquinolones, was initially attributed to continuous induction of the SOS response and the concomitant expression of the SfiA protein, which is an inhibitor of cell division [53]. However, it has been demonstrated that persistent DNA damage generated by the fluoroquinolones can be repaired by a mutagenic mechanism that is dependent on induction of the SOS response [54]. Consequently, mutagenic repair of quinolone-induced DNA damage is recognized as a potential mechanism that can facilitate development of quinolone-resistant strains [55,56].

Since ribosome inhibitors are very efficient in selectively inhibiting global protein synthesis in bacteria, they could be used *in lieu* of DSBR-inhibiting compounds, to robustly enhance the susceptibility of *M. smegmatis*, and other bacteria with multiple DSBR pathways, to DSB-inducing drugs. Moreover, the use of protein inhibitors in combination with DSB-inducing compounds could be used for treatment of infections caused by bacteria that utilizes both homologous recombination and NHEJ for repair of DSBs in actively growing cells. Evidently, the rationale of this chemotherapeutic strategy is to inhibit the synthesis of DSBR proteins within the bacterial cell in response to the DSBs in order to enhance their susceptibility to DSB-inducing drugs. The use of ribosome inhibitors together with DSB-inducing drugs might also be useful for resuscitating some of these individual drugs, which are currently obsolete in clinic due to development of resistance against their primary cellular target.

## Preliminary data from ongoing study

**Figure 4.**
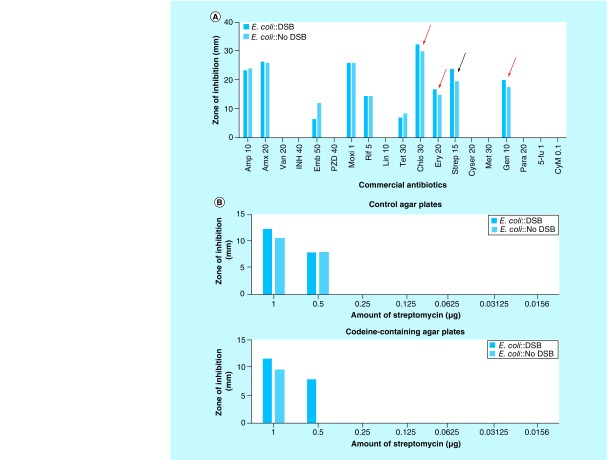
Interaction of streptomycin with codeine enhances the susceptibility of *Escherichia coli* to DNA double-strand breaks. **(A)** Profile of selected commercial antibiotics against *Escherichia coli* subjected (or not) to DNA double-strand breaks. Arrows indicate commercial antibiotics that modestly increased the sensitivity of *E. coli* to DNA double-strand breaks. These antibiotics are chloramphenicol (30 μg/μl), erythromycin (20 μg/μl), streptomycin (15 μg/μl) and gentamicin (10 μg/μl). **(B)** MIC determination for streptomycin on agar plates containing (or not) codeine. *Escherichia coli*::double-strand break represents an *E. coli* strain containing a system that induces a site-specific DNA double-strand break at the *lacZ* locus of the chromosome [9]. *Escherichia coli*::NO double-strand break represents the control *E. coli* strain, which is unable to induce the site-specific DNA double-strand break at the *lacZ* locus. DSB: DNA double-strand break; MIC: Minimum inhibitory concentration.

Preliminary data indicate that constitutive induction of a site-specific DSB in a repair-proficient *E. coli* strain [9] leads to an increase in sensitivity to ribosome-targeted antibiotics (chloramphenicol, erythromycin, streptomycin and gentamicin; arrows of Figure 4A). Despite the enhanced sensitivity to this class of antibiotics, the MIC of streptomycin was unaffected in the presence of DSB induction relative to the *E. coli* cells that were not subjected to DSB induction (Figure 4B; control agar plate). This observation implies that inhibition of global protein synthesis by streptomycin elicits marginal synergy with DSBs against *E. coli*. However, the synergy is boosted following addition of a number of well-known compounds (such as codeine), which led to a decrease in the MIC of streptomycin against *E. coli* subjected to constitutive induction of DSBs. The modes of action of codeine that resulted in the lower MIC of streptomycin in the presence of DSB induction are currently unknown and are being investigated. It is also unknown whether this biological phenomenon is unique to *E. coli* or can be exhibited by other pathogenic bacteria. The ongoing work has also identified significant number of fungal extracts falling to the two groups of compounds that fit the expectations described in Figures 1–4. The important work of isolating the specific compounds is earnestly underway, the success of which will yield vital new class of antibiotics with new mechanisms of action to combat drug-resistant infection.

## Conclusion & future perspective

DSB formation and inhibition of the concomitant repair event represent distinct molecular mechanisms that could be targeted for the development of combination chemotherapy against bacterial infections. Homologous recombination is predominantly used for repairing DSBs in most bacteria, hence it stands out as the preferred pathway whose inhibition could render these bacteria extremely susceptible to DSB-inducing drugs. For bacteria that utilize multiple pathways for repairing DSBs, inhibition of global protein synthesis could be targeted to deplete the DSBR proteins in order to enhance the susceptibility of these bacteria to DSB-inducing drugs.

On the basis of the concepts and approaches discussed in this perspective, it is envisaged that significant advances will be attained in the discovery and development of novel antimicrobial compounds targeting genome stability. The use of omics can provide insight on the holistic regulatory landscape for DSB formation and repair. Cellular factors that modulate the formation and repair of DSBs would be vital novel targets for development of robust antibiotic chemotherapy. Screening of natural products and libraries of synthetic derivatives would also lead to identification of novel compounds that exhibit antimicrobial activity via formation of persistent DSBs and inhibition of the concomitant repair event. Evidence will be reported in literature to explain in great detail the basis for synergy of specific small molecules that generate DSBs and inhibit the repair in the ESKAPE pathogens (***E**nterococcus faecium*, ***S**taphylococcus aureus*, ***K**lebsiella pneumoniae*, ***A**cinetobacter baumanni*i, ***P**seudomonas aeruginosa*, ***E**nterobacter* spp.) and fluoroquinolone-resistant bacteria. These achievements would propel preclinical studies to validate drug candidates that were selected on the basis of compelling evidence from chemical biology. The use of different animal models for pharmacodynamics would highlight the efficacy of this potential combination chemotherapy and might possibly reveal pitfalls that were not addressed during the preceding *in vitro*/*in vivo* studies. Ultimately, drug combinations that exhibit synergistic effect against drug-resistant infections are anticipated to be developed and they should be robust against development of drug resistance.

**Executive summary**

This article discusses a strategy for the development of robust chemotherapy against antimicrobial-resistant infections by:

- Providing insights into the possibility of exploiting the bacterial double-strand break (DSB) repair pathways as targets.
- Exploring crucial synergistic combinations between DSB-inducing compounds and inhibitors of DSB repair.
- Examining the effectiveness of this new therapeutic regimen in combating antimicrobial-resistant infections.

The proposed chemotherapeutic strategy is discussed as a perspective, highlighting the possibility of utilizing bacterial DSB repair pathways as targets for the discovery and development of novel antibiotics.
